# Advancements in the Management of Fragility Fractures in Orthopaedic Patients

**DOI:** 10.7759/cureus.74065

**Published:** 2024-11-20

**Authors:** Saskia Locke, James Doonan, Bryn Jones

**Affiliations:** 1 Orthopaedics, Glasgow Royal Infirmary, Glasgow, GBR

**Keywords:** clinical frailty scale, fragility fractures, frailty, locking plate fixation, minimally invasive plate osteosynthesis (mipo), osteoporosis, pelvic fractures, peripheral nerve block, regional anaesthesia, vertebral fracture

## Abstract

Osteoporosis is a major risk factor for fragility fractures. The British Orthopaedics Association Standards for Trauma and Orthopaedics (BOAST) and Getting it Right First Time (GIRFT) guidelines on fragility fracture management highlight the need to initiate prompt, coordinated multidisciplinary care with a focus on early mobilisation to improve patient outcomes. Medical management of fragility fractures focuses on the prevention of progressive frailty. Advancements in medical therapy include romosozumab, recommended by the National Institute for Health and Care Excellence guidance in patients with imminent fracture risk, which improves overall bone mineral density. Regional nerve blocks are an increasingly common form of perioperative anaesthesia with fewer side effects than opioids and rates of postoperative delirium. Surgical management of osteoporotic fractures poses unique challenges, such as complex fracture patterns and increased risk of implant failure. The surgical approach to fragility fractures has undergone major advancements over the past 20 years, with developments such as polyaxial locking and far cortical locking systems that achieve secondary bone healing, as well as cement augmented screw fixation to provide stable fixation in osteoporotic bone. The development of minimally invasive surgical approaches has led to improved periosteal blood flow around a fracture site, as well as reduced operating time, hospital stay, and time to pain-free weight-bearing. In the future, we are likely to see a focus on minimally invasive surgical techniques for vertebral and pelvic fragility fractures to improve patients' mobility and independence before discharge, subsequently improving quality of life and preventing progressive frailty.

## Introduction and background

Fragility fractures are defined as fractures that occur from low-energy trauma, specifically trauma that is equivalent to falling from standing height or less, with the majority of fractures occurring in the vertebra, proximal femur, or distal radius [1,2]. Osteoporosis, as defined by a bone mineral density (BMD) of 2.5 standard deviations below the reference mean, is a major risk factor for fragility fractures [2,3]. Over three million people in the United Kingdom have osteoporosis, with the estimated total annual cost of fragility fractures being £4.4 billion, including social care costs. Risk factors include increasing age, female sex, poor nutrition, menopause, smoking, poor mobility, and long-term use of corticosteroid medications [1,2,4]. Neck of femur fractures have been shown to be associated with a significantly increased one-year postoperative mortality of 27.3%, which is three times that of the general population [5].

Current guidance on fragility fracture management

The British Orthopaedics Association Standards for Trauma and Orthopaedics (BOAST) published specific guidance on the care of frail orthopaedic trauma patients in 2019. This emphasised the need for coordinated, multidisciplinary care of patients with fragility fractures, as well as major trauma patients with a clinical frailty scale of five or above. This multidisciplinary approach draws on the expertise of orthogeriatricians, anaesthetists, physiotherapists, and surgeons to assess for delirium and ensure prevention, consider the early use of regional anaesthesia as part of the patient's pain management, as well as the need to facilitate full weight-bearing in these patients within 36 hours of admission [6,7].

The national multidisciplinary guidance on non-ambulatory fragility fracture management has been published by Getting it Right First Time (GIRFT) [8]. This guidance, published in May 2024, builds on the advancements made in hip fracture care and adapts these to all fragility fractures impairing mobility [6,8,9]. GIRFT advises that the aim of facilitating weight-bearing within 36 hours of admission through surgical intervention should be applied to all non-ambulatory fragility fractures [6,8]. The GIRFT guidance also appeals for joint care between surgeons, physicians, and physiotherapists, with holistic care, including a fall assessment and bone health review during admission [8]. The guidance specifies that reviews of mobilisation, analgesia, delirium, nutrition, and hydration should be carried out daily, which is more rigorous than earlier national guidance [6,8].

## Review

Medical management of fragility fractures

Treatment of Osteoporosis

BOAST and GIRFT guidance on the management of patients with fragility fractures includes a bone health review with appropriate medical treatment if required [6,8]. Medical management includes performing a dual-energy X-ray absorptiometry (DEXA) scan to obtain a BMD T-score and the management of risk factors such as smoking and deficiencies in vitamin D and calcium [2]. Combined vitamin D and calcium supplementation has been shown to reduce fracture risk by 6% and reduce hip fracture risk by 16% when compared with no supplementation [10]. Initiation of a bisphosphonate such as alendronic acid is advised if the T-score is below -2.5 [11].

The osteoclast inhibitor and monoclonal antibody, denosumab, has been the mainstay of treatment for patients who may not tolerate alendronic acid and have a low T-score in combination with other risk factors for fragility fractures (parental history of hip fracture, alcohol intake of 4 or more units per day, and rheumatoid arthritis) [12]. It has been shown to be an effective agent in treating bone loss; however, it is important to be aware of a peak incidence in vertebral fractures, or 'rebound fractures' at nine months of stopping denosumab therapy. This risk is significantly reduced with subsequent alendronate (p = 0.034) or zoledronic acid (p = 0.037) treatment at the time of stopping denosumab [13].

In May 2022, the National Institute for Health and Care Excellence (NICE) published recommendations on the use of a sclerostin-binding monoclonal antibody, romosozumab, which has been shown to be effective in increasing the lumbar spine BMD over 12 months by 12.5% in comparison to an increase of 7.2% in patients taking denosumab [14]. Romosozumab is recommended in patients with imminent fracture risk, identified as those with a history of major osteoporotic fractures (such as spine, hip, radius, or humerus fractures) within the last 24 months. Treatment with romosozumab involves monthly subcutaneous injections for 12 months, followed by long-term treatment with alendronic acid [14,15].

Analgesia for Patients With Fragility Fractures

Both pain and perioperative opioid use are risk factors for delirium [16]. If opioids are to be used in the elderly, a reduced dose should be used, and side effects such as constipation and nausea should be anticipated and treated promptly [17]. Regional nerve blocks are an increasingly common form of analgesia used in orthopaedic patients, and they have been shown to reduce the rates of postoperative delirium in patients without cognitive impairment in multiple meta-analyses [16,18,19]. Regional nerve blocks reduce the amount of opioids required for an adequate analgesic effect. They can be carried out by anaesthetists preoperatively; however, femoral nerve and fascia iliaca compartment blocks are frequently performed by emergency physicians on admission for hip fracture patients and have been adopted as a recommendation in Scottish hip fracture care [20,21]. Not only have peripheral nerve blocks been shown to reduce delirium in the perioperative period, but they have also been shown to reduce the risk of pneumonia and time to first mobilisation following surgery [22].

Surgical management of fragility fractures

Osteoporotic fractures result in complex fracture patterns, such as metaphyseal comminution and involvement of the tuberosities around the hip and shoulder, as well as impaction of fracture fragments [23]. Previous conventional fixation methods focussing on screw purchase for rigid fracture fixation and primary bone healing may lead to implant failure, as the reduced BMD limits the degree of compression, resulting in friction, and torsional strength achieved between the screws and the bone [24,25]. BOAST guidance emphasises that surgery should be performed with the intention of restoring full weight-bearing for activities of daily living and within 36 hours of admission [6]. Surgical systems that have been developed to aid bone healing and restore patient function include hybrid locking and non-locking plate systems, far cortical locking (FCL) screws, polyaxial locking screws, and cement-augmented screw fixation [25-28]. Further consideration into reducing muscle detachment and disruption of periosteal blood supply at the fracture site has led to the development of minimally invasive plate osteosynthesis (MIPO) approaches in osteoporotic femoral, tibial, and humeral fractures [29], which are summarised in Table 1.

**Table 1:** Plate fixation system advancements in the management of fragility fractures.

Surgical Advancement	Features	Advantages
Hybrid locking and non-locking plate systems [25,32]	A combination of both locking and conventional non-locking screws.	Locking screws add strength to the plating system, whilst non-locking screws achieve a reduction of the bone to the plate.
Polyaxial locking systems [25–27,34]	Allow variable screw angles to achieve different screw trajectories in relation to the plate.	Polyaxial locking systems increase the spread of load, reducing the risk of stress fractures. They also allow the surgeon to adapt screw angles based on the fracture pattern and position around a prosthesis.
Far cortical locking (FCL) screws [30,36,38,39]	Screw shafts are flexible as they have a reduced diameter, and they have a threaded tip that engages the far cortex. FCL screws provide near cortex motion control with a non-threaded collar featuring a slightly increased diameter compared to the shaft.	FCL screws allow for a degree of micromotion around the fracture site, encouraging callus formation and secondary bone healing. Progressive stiffening is achieved, with maximum stiffness gained once a callus is formed around the near cortex shaft.
Minimally invasive plate osteosynthesis (MIPO) [29,32,41,46-48]	Minimal length of wounds with proximal and distal incisions to allow insertion and fixation of a large plate.	MIPO techniques reduce the extent of muscle detachment and any compromise in periosteal blood supply at the fracture site.
Cement-augmented screw fixation [28,42,43,45]	Injection of bone cement in the planned trajectory of the screw, followed by screw insertion.	The cement provides a strong bone-cement interface that achieves stable fixation in osteoporotic bone.

Hybrid Plating Systems

Locking plates and screws act as a fixed-angle construct, ensuring that compressive forces are distributed across all locked screws, which osteoporotic bone is more able to withstand, reducing the risk of implant failure [25]. These locking plate systems also have some disadvantages depending on how the device is used: increasing the number of screws can increase fracture rigidity, reducing micromotion leading to a reduction in secondary bone healing, and the added fracture stiffness can increase the risk of periprosthetic fractures [30,31]. Hybrid plating systems allow for a combination of both locking and conventional non-locking screws. The non-locking screws achieve a reduction of the plate to the bone and some micromotion of fracture fragments, and the locking screws add strength to the plating system. It is important to note that at least three bicortical locking screws are required on either side of the fracture site to improve torsional stiffness in osteoporotic bone. Locking screws placed between the fracture site and non-locking screws prevent the non-locking screws from loosening [25]. Hybrid plating systems have also been shown to achieve higher torsional strength and only slightly reduced axial strength in osteoporotic diaphyseal fracture models [32].

Polyaxial Locking Systems

Polyaxial locking systems allow variable screw angles to achieve different screw trajectories in relation to the plate. This allows the surgeon to adapt the screw angles based on the fracture pattern, as well as provide a solution when placing screws around a prosthesis or internal fixation device in periprosthetic fractures [33]. These systems have also been shown to increase the spread of the load, reducing the risk of stress fractures [23,33]. When examining the effect of polyaxial locking fixation systems on patient outcomes, they have been shown to improve patient-reported scoring systems in proximal humerus fractures, as Königshausen et al. achieved a Constant score of 78% on the injured side when compared to contralateral uninjured arm (mean injured arm Constant score 66.0 compared to 84.6 on the healthy side) [34]. Periprosthetic-specific polyaxial plates have also shown promising rates of bone healing in periprosthetic femoral fractures around stemmed total knee replacements in a case series carried out by Gonzalez-Morgado et al.; however, high rates of complications are to be expected in multimorbid and frail patients [35].

Far Cortical Locking Screws

FCL technology has been shown to improve bone union rates due to the promotion of flexible fixation and, therefore, secondary bone healing [26,36]. FCL screw shafts are flexible as they have a reduced diameter and a threaded tip that engages the far cortex. They also provide near cortex motion control with a non-threaded collar featuring a slightly increased diameter compared to the shaft. This allows for a degree of micromotion around the fracture site, encouraging callus formation and secondary bone healing. Progressive stiffness is achieved, with maximum stiffness gained once a callus is formed around the near cortex shaft [37]. FCL technology has been shown to improve bone union rate in proximal humeral fracture significantly at 12 weeks, with 94.4% bone union in the FCL group compared to 66.7% bone union in the conventional bicortical locking screw group [36]. The constructs have also been tested in distal femur fractures and periarticular knee fractures, and this has shown effective fixation with minimal complication rates [38,39].

Minimally Invasive Periosteal Osteosynthesis

MIPO techniques feature the use of proximal and distal, and sometimes midshaft incisions, to allow insertion and fixation of a large plate [31]. The aim of MIPO is to reduce the extent of muscle detachment with the aim to preserve periosteal blood supply, reduce wound complications, and improve rehabilitation [31]. The technique has shown promising results in small studies of upper limb fractures [29,40,41]. A medial MIPO technique for humeral shaft fractures has also been shown to achieve bone union in all patients and provided a promising, safe approach in terms of reducing iatrogenic radial nerve injury rates in a case series of 21 patients [40]. It is important to note that although MIPO reduces operative time, the intraoperative fluoroscopy time has been shown to be higher when using a MIPO approach in humeral shaft fractures [41].

Cement-Augmented Screw Fixation

Cement-augmented screw fixation has been developed to improve fixation strength in bone with a low BMD [28]. This method involves the injection of bone cement into where the screw will be sited, followed by prompt insertion of the bone screw under X-ray guidance, whilst also assessing for bone cement leakage [42]. The method has initially been shown to be effective in the posterior stabilisation of osteoporotic spines; however, a recent study by Peng et al. has shown that there are no significant differences in screw loosening rates between a cement-augmented screw group and a conventional pedicle screw group of patients for isthmic spondylolisthesis, and cement augmentation led to increased operative time and intraoperative blood loss [43,44]. Despite this, the technique has also been used in a polyaxial locking system in proximal humerus fractures with significantly lower rates of complications such as screw cut out and failure of the fixation [45]. As evidence is contrasting, and there are serious complications of cement-augmented screw fixation, such as cement leakage, pulmonary cement embolisation, and challenging revision surgery, further work is needed to determine which patients would benefit [28,43].

Vertebral and Pelvic Fragility Fractures

Vertebral fragility fractures are the most common osteoporotic fracture. In patients with no red flag features (lower limb weakness, altered sensation, radicular pain, or an unstable fracture), vertebral fractures are conservatively managed with strong analgesia with a focus on avoiding prolonged bed rest [49]. NICE recommends surgical management of a recent unhealed compression fracture after a period of unsuccessful conservative management where the location of pain is congruent with the level of confirmed fracture on clinical examination and imaging [50]. Surgical management of vertebral fragility fractures is by either injection of bone cement into the fractured vertebra to improve stability (percutaneous vertebroplasty) or inflation of a balloon to increase the vertebral height prior to bone cement injection (percutaneous balloon kyphoplasty) [49,51]. As with cement-augmented screw fixation, these procedures have been shown to lead to cement leakages and extravasation, as well as a reduction in vertebral height following balloon removal. Further development has led to the insertion of stents in order to maintain a restored vertebral height (percutaneous vertebral augmentation technique) [51].

A 90-day mortality in non-operatively managed pelvic fracture patients over the age of 70 has been shown to be up to 11 times that of the general population. This high level of mortality is equivalent to that found in hip fracture patients [48]. Whilst the widely accepted management of fragility fractures of the pelvis is non-operative, involving analgesia and early mobilisation, there is a movement to study operative interventions with the aim to reduce the period of immobility and improve independence before discharge [48,52]. These interventions can be subdivided into posterior and anterior pelvic stabilisation methods, with anterior fixation rarely occurring in isolation [53]. Posterior stabilisation methods include percutaneous sacro-iliac screw fixation, the use of sacral bars, and the use of cement to perform sacroplasty [52,54]. Anterior fixation tends to be achieved with either percutaneous screws or open reduction and plate fixation. A systematic review by Wilson et al. suggests operative management of pelvic fractures after a brief period of failed conservative management to avoid morbidity associated with immobility [52].

## Conclusions

The treatment of fragility fractures relies on the coordination of advances in medical management and surgical techniques. Whilst surgical management focuses on the prevention of further fractures via good fracture fixation and early mobilisation of patients to prevent progressive frailty, medical management uses DEXA scanning to highlight those who require further treatment with bisphosphonates and biologic therapies to improve overall bone health.

The need to facilitate full weight-bearing in frail patients within 36 hours of admission is widely accepted amongst orthopaedic surgeons. This objective guides surgical planning and decision-making, aiming for reduced operative time and adequate fracture reduction for the patient. There is now an increasing evidence base for newer surgical approaches to fix complex osteoporotic fractures that focus on multiple factors such as stability of fracture fixation, quality of bone healing, and the preservation of periosteal blood supply to facilitate optimal healing. These methods and fixation systems achieve improved fracture unions at follow-up and improved patient-reported outcome scores for complex osteoporotic fractures. Management of pelvic and vertebral fractures is mostly conservative, with surgical management often being challenging or leading to complications such as extravasation of cement in vertebroplasties. Future work should focus on creating a stronger evidence base investigating the minimally invasive surgical management of patients with fragility fractures of the spine or pelvis that fail conservative management. This could lead to more treatment options to reduce pain and improve mobility and independence in these patients, potentially reducing hospital admission time and relieving pressure from the social care system.
